# T Cells in Vascular Inflammatory Diseases

**DOI:** 10.3389/fimmu.2014.00504

**Published:** 2014-10-14

**Authors:** Lucas L. Lintermans, Coen A. Stegeman, Peter Heeringa, Wayel H. Abdulahad

**Affiliations:** ^1^Department of Rheumatology and Clinical Immunology, University of Groningen, University Medical Center Groningen, Groningen, Netherlands; ^2^Department of Nephrology, University of Groningen, University Medical Center Groningen, Groningen, Netherlands; ^3^Department of Pathology and Medical Biology, University of Groningen, University Medical Center Groningen, Groningen, Netherlands

**Keywords:** vascular inflammation, ANCA-associated vasculitis, atherosclerosis, T lymphocytes, effector memory T cells, Kv1.3 channels

## Abstract

Inflammation of the human vasculature is a manifestation of many different diseases ranging from systemic autoimmune diseases to chronic inflammatory diseases, in which multiple types of immune cells are involved. For both autoimmune diseases and chronic inflammatory diseases several observations support a key role for T lymphocytes in these disease pathologies, but the underlying mechanisms are poorly understood. Previous studies in several autoimmune diseases have demonstrated a significant role for a specific subset of CD4^+^ T cells termed effector memory T (T_EM_) cells. This expanded population of T_EM_ cells may contribute to tissue injury and disease progression. These cells exert multiple pro-inflammatory functions through the release of effector cytokines. Many of these cytokines have been detected in the inflammatory lesions and participate in the vasculitic reaction, contributing to recruitment of macrophages, neutrophils, dendritic cells, natural killer cells, B cells, and T cells. In addition, functional impairment of regulatory T cells paralyzes anti-inflammatory effects in vasculitic disorders. Interestingly, activation of T_EM_ cells is uniquely dependent on the voltage-gated potassium Kv1.3 channel providing an anchor for specific drug targeting. In this review, we focus on the CD4^+^ T cells in the context of vascular inflammation and describe the evidence supporting the role of different T cell subsets in vascular inflammation. Selective targeting of pathogenic T_EM_ cells might enable a more tailored therapeutic approach that avoids unwanted adverse side effects of generalized immunosuppression by modulating the effector functions of T cell responses to inhibit the development of vascular inflammation.

## Introduction

Vasculitides comprises a group of rare diseases, characterized by inflammation of the blood vessel walls. The clinical manifestations are dependent upon the localization, the type of vessel involved as well as the nature of the inflammatory process. Vasculitis constitutes, in most cases, as a primary autoimmune disorder, but can also be secondary to other conditions. The underlying conditions to secondary vasculitis are infectious diseases, connective tissue disorders, or hypersensitivity disorders. In general, primary vasculitides are systemic diseases with variable clinical manifestations making it difficult to classify. According to the latest Chapel Hill Consensus Conference, primary systemic vasculitides can be divided into seven main entities of which three are most common; large vessel vasculitis, medium vessel vasculitis (MVV), and small vessel vasculitis (SVV) (1). The group of large vessel vasculitides (LVV) affects the aorta and its major branches. The two major variants of LVV are giant cell arteritis (GCA) and Takayasu’s arteritis (TA). MVV is vasculitis that predominantly affects medium arteries defined as the main visceral arteries and their branches. The two major categories are polyarteritis nodosa (PAN) and Kawasaki disease (KD). SVV is divided into anti-neutrophil cytoplasmic antibody (ANCA)-associated vasculitis (AAV) and immune complex SVV (2). AAV is characterized by necrotizing vasculitis with few or no immune deposits that predominantly affects small vessels, which lead to systemic organ damage. AAV are associated with the presence of circulating ANCA that are directed against proteinase-3 (PR3) or myeloperoxidase (MPO), proteins in the cytoplasmic granules of neutrophils. This group of systemic vasculitis includes granulomatosis with polyangiitis (GPA), primarily associated with antibodies to PR3–ANCA and microscopic polyangiitis (MPA) and eosinophilic granulomatosis with polyangiitis (EGPA), both principally associated with antibodies to MPO–ANCA.

Besides autoimmune disorders related to vascular inflammation, a more common chronic vascular inflammatory disease is atherosclerosis. Clinical evidence indicates that patients suffering from large and medium-sized vessel vasculitis show accelerated atherosclerosis (3). In SVV, this relation is less well defined. However, many patients with SVV carry several risk factors (e.g., impaired renal function, persistent proteinuria, and increased level of C-reactive protein) that contribute to the acceleration of the atherosclerotic process (3, 4). Enhanced oxidation processes, persistently activated T cells and reduced numbers of regulatory T (T_REG_) cells are among the many pathophysiological factors that play a role in the acceleration of atherogenesis (5). Both vasculitis and atherosclerosis, although in nature different forms of chronic conditions, reveal similarities in T cell repertoire that occur within the process of vascular inflammation.

This review provides an overview of the role of adaptive immune mechanisms in vascular inflammation focusing on the T lymphocytes in particular. The main emphasis will be on the role of effector memory T (T_EM_) cells in vasculitis (i.e., AAV and atherosclerosis) and the potential therapeutic interventions for modulating the activity of these cells.

## T Lymphocytes: Key Participants in Vascular Inflammation

T cells are recruited to the vessel wall in conjunction with macrophages, but in lesser quantity. In the blood vessel wall or tissues, T cell responses are initiated by signals generated via the association of TCR complexes with specific peptide–MHC protein complexes on the surface of antigen-presenting cells (APCs) and through signals provided by co-stimulators expressed on APCs. The responses to antigen and co-stimulators include synthesis of pro-inflammatory mediators (e.g., IFN-γ) cellular proliferation, differentiation into effector and memory cells, and performance of effector functions. These initial events further amplify the inflammatory response, aggravating disease progression. Different T cell subsets exist that can influence vascular inflammation in various ways. In the last decade, substantial progress has been made in the characterization of T cell mediated responses in vascular inflammation.

### T cell involvement in AAV and atherosclerosis

In AAV it has been postulated that ANCA *in vivo* bind to surface expressed auto-antigens (PR3 or MPO) on primed neutrophils, which subsequently activates the neutrophils (6). These activated neutrophils enhance neutrophil degranulation and the release of cytotoxic products that promote endothelial cells damage leading to vascular inflammation and injury (6). This initial inflammatory response mediated by the innate immune system creates a pro-inflammatory (micro)environment to attract cells from the adaptive immune system. In the case of autoimmune mediated vascular pathologies, like AAV, loss of self-tolerance, and continuous antigen presentation also contributes to the involvement of the adaptive immune system. The contribution of T cell mediated immune responses in vascular inflammation is most likely because infiltrating T cells are detected in inflammatory lesions observed in the microvascular bed of kidney, lung, and in nasal biopsies from AAV patients (7–11). In accordance with these findings, soluble T cell activation markers [soluble interleukin-2-receptor (sIL-2R) and soluble CD30] are elevated in plasma or serum and have been shown to be associated with disease activity in AAV (12–15). Also, ANCA antigen specific T cells have been detected in AAV (16, 17). Moreover, the IgG subclass distribution of ANCA, predominantly consisting of IgG1 and IgG4 implies isotype switching of ANCA for which T cells are required (18). Importantly, Ruth et al. demonstrated a pivotal role of T cells in the expression of crescentic glomerulonephritis (19). They induced experimental anti-MPO-associated crescentic glomerulonephritis by immunizing C57BL/6 mice with human MPO followed by subsequent challenge with anti-glomerular basement membrane (anti-GBM) antibodies. Mice depleted of T cells at the time of administration of anti-GBM antibodies developed significantly less glomerular crescent formation and displayed less cell influx in glomeruli compared with control mice. Interestingly, specific T cell depleting therapies with anti-CD52 antibodies (Alemtuzumab) or anti-thymocyte globulin can induce remission in refractory AAV patients (20, 21).

Atherosclerosis is considered a chronic inflammatory disease, characterized by a slowly progressing passive lipid accumulation in large and medium-sized blood vessels that ultimately leads to the formation of plaques. Both innate and adaptive immunity are involved in this process. Ait-Oufella et al. recently reviewed the role of the adaptive immune response in atherosclerosis and discussed the role of dendritic cells (DCs) in the control of T cell involvement in atherosclerosis (5). Classically, DCs accumulate in the atherosclerotic plaque through direct chemokine mediated recruitment. DCs take up (atherosclerotic-specific) antigens such as ApoB100 and LDL and become activated and mature. Subsequently, DCs migrate to draining lymph nodes, where they can present antigens to naïve T cells. After activation, these T cells develop into effector cells, clonally expand and enter the bloodstream. When effector T cells are recruited into atherosclerotic plaques they are reactivated by antigens presented by local macrophages and DCs, boosting the immune response. In human atherosclerotic lesions, the ratio of macrophages to T cell has been reported to be approximately 10:1, thus T cells are not as abundant as macrophages. However, because T cells are activated in the lesions resulting in the production of pro-atherogenic mediators, they can importantly contribute to lesion growth and disease aggravation. The first evidence of T cell involvement in atherosclerosis came with the demonstration that MHC class II positive cells and T cell cytokines (e.g., IFN-γ) are expressed in human atherosclerotic plaques (22). Later, the presence of T cells was observed in atherosclerotic plaques in humans (23, 24) and mice (25, 26). These observations only demonstrated the association of T cell with atherosclerosis but did not revealed the role of T cells in atherogenesis. However, Zhou et al. demonstrated a specific role of T cells in atherogenesis using an animal model of atherosclerosis. They showed that transfer of CD4^+^ T cells into ApoE^−/−^ mice crossed with immunodeficient mice (scid/scid mice) fully reversed the atheroprotection provided by T and B cell deficiency (27).

**Figure 1:**
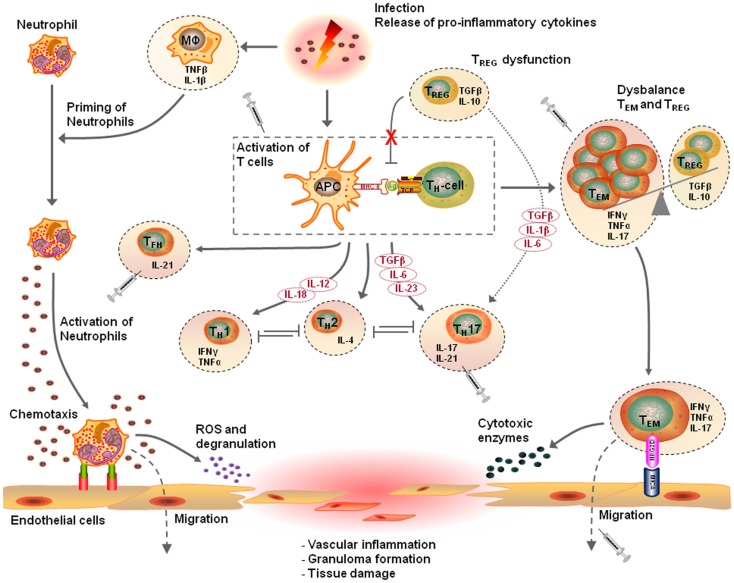
**Proposed pathophysiological mechanism of T cell mediated vascular inflammation**. Vascular inflammation is initiated by a pro-inflammatory trigger such as an infection. Release of pro-inflammatory cytokines causes priming of neutrophils, up-regulation of adhesion molecules on endothelial cells, and an expansion of circulation effector T cells. Activation of primed neutrophils enhances vessel wall adherence and the transmigration capacity of the neutrophils. Production of reactive oxygen species and degranulation of fully activated primed neutrophils causes damage to vascular endothelial cells. This acute injury together with pro-inflammatory triggers elicits an innate inflammatory response that recruits T lymphocytes, which replace the neutrophils and either resolves or mediate the development of vasculitis. In this pro-inflammatory environment, the innate immune system with antigen-presenting cells (APCs) and T cells start to mediate the inflammatory response. Distinct cytokine patterns in combination with a defect in regulatory T (T_REG_) cell function or frequency results in expansion of effector memory T (T_EM_) cells. The dysbalance in the homeostasis of T_REG_ cells and T_EM_ cells, results in additional releases of pro-inflammatory cytokines promoting neutrophil priming and persistent activation of T_EM_ cells. Expanded circulating T_EM_ cells upregulate their killer immunoglobulin-like receptor (NKG2D) and interact with their ligand major histocompatibility complex class-I chain-related molecule A (MICA) on vascular endothelial cells. This event results in the migration of T_EM_ cells into target tissues, drive granuloma formation leading to tissues destruction in a perforin-dependent, and granzyme-dependent way, ending up in vasculitis. The T cell driven vascular inflammatory response is a multistep process and has different therapeutic possibilities. For this purpose, selective T_EM_ cell modulation might be beneficial to regulate the T_EM_ cell activity, proliferation, and migration. Other therapeutic options are modulation of T cell activation by interfering with co-stimulatory molecules, depletion of T cells, inhibition of T cell migration, or neutralizing secreted pro-inflammatory cytokines (This figure was created using Visi ScienceSlides^®^ Software).

Taken together, these observations indicate that T cell mediated immunity is an important contributor to the pathogenesis of vascular diseases such as AAV and atherosclerosis. In line with this, different T cell populations have been identified in vascular inflammation as will be discussed below. Figure 1 presents a proposed mechanism of the T cell mediated vascular inflammatory process.

### T helper cells in vascular inflammation

Aberrant T helper (T_H_) cell polarization has been described in patients with vascular diseases. The involvement of different T_H_ cells subsets in the pathogenesis of vascular disease has been suggested to depend on disease activity/stage and whether the disease is localized or systemic.

In AAV, analysis of patients sera for soluble markers associated with either T_H_1 cells (IFN-γ, sCD26) or T_H_2 cells (IL-4, IL-5, IL-10, IL-13, sCD23, and sCD30) revealed a shift toward a T_H_2-type response in patients with active generalized disease, whereas a T_H_1-type response is predominant in patients with localized disease (28, 29). Consistent with these observations, analysis of nasal granulomatous lesions from AAV patients demonstrated a relative increase of cells expressing T_H_1-associated markers such as IFN-γ and CD26 during localized disease, whereas the T_H_2-associated marker IL-4 was found in generalized AAV (11). In addition, Lamprecht et al. compared chemokine receptors on peripheral blood-derived T cells. The inducible inflammatory T_H_1-type chemokine receptor CCR5 was more prominent in the granulomatous lesions of AAV patients (30).

Similar to AAV, a study on cytokine expression in advanced human atherosclerotic plaques confirmed the dominance of pro-inflammatory T_H_1 cytokines (IFN-γ, TNF-α, and IL-2) (31). Genetic deficiency in IFN-γ or its receptor in ApoE^−/−^ mice reduced atherosclerotic lesion formation and enhanced plaque stability (32), whereas exogenously administered IFN-γ enhanced atherosclerosis in ApoE^−/−^ mice (33). Intriguingly, it seems that the protective effect of IFN-γ deficiency is restricted to male ApoE^−/−^ mice (34). In addition, several studies revealed that intervention in IL-12 or IL-18 gene, or receptor function was found to reduce plaque development in mouse models of atherosclerosis (35–37). Furthermore, administration of these cytokines accelerated disease progression (38, 39). Collectively, these data point toward a pro-inflammatory T_H_1 response in atherosclerosis. However, the role of T_H_2 immune responses in atherosclerosis is controversial. IL-4, the signature cytokine of the T_H_2 lineage, is not frequently observed in human atherosclerotic plaques (31). Moreover, experimental studies examining the involvement of T_H_2 cells are contradictory, some showing pro-atherosclerotic effects (36, 40), whereas others show no or athero-protective effects (41, 42).

Overall, the balance between T_H_1 and T_H_2 cells plays a key role in the development of vascular inflammation. Interestingly, in the last decade T_H_17 cells have emerged as a new CD4^+^ T cell subset characterized by secretion of IL-17A and other cytokines including IL-17F, IL-21, and IL-22. These cells are considered another major pathogenic effector subset involved in the development of inflammatory and autoimmune diseases (43).

IL-17 has been reported to promote the release of the pro-inflammatory cytokines IL-1β and TNF-α from macrophages (44), which are essential for priming and activation of neutrophils. Furthermore, this pro-inflammatory milieu induces CXC chemokine release (45) and up-regulation of endothelial adhesion molecules (46) responsible for the recruitment of neutrophils to the site of inflammation (47). These pro-inflammatory events suggest that IL-17 may directly contribute to the acute vascular inflammatory response in AAV. Convincing experimental evidence that support this notion comes from several studies. Hoshino et al. demonstrated that neutrophils produce IL-17A and IL-23 in response to MPO–ANCA creating local conditions to promote T_H_17-mediated autoimmunity (48). In addition, Gan et al. showed that immunization of C57BL/6 mice with murine MPO resulted in MPO-specific dermal delayed type hypersensitivity and systemic IL-17A production (49). Upon injection of low-dose anti-GBM antibodies these mice developed glomerulonephritis. In contrast, IL-17A deficient mice were nearly completely protected from disease induction due to reduced neutrophil recruitment and MPO deposition (49). Consistent with this finding, Odobasic et al. demonstrated that IL-17A contributes to early glomerular injury, but it paradoxically, attenuates the severity of fully established crescentic disease by limiting the T_H_1 responses (50). They used a mouse model of crescentic anti-GBM glomerulonephritis assessing the renal injury and immune responses in IL-17A^−/−^ and in wild-type (WT) mice. Crescentic glomerulonephritis was enhanced in IL-17A^−/−^ mice, with increased glomerular T cell accumulation and augmented T_H_1 responses (50). In contrast, mice lacking IL-12(p35), the key T_H_1-promoting cytokine, had decreased T_H_1 responses and increased T_H_17 responses and developed less severe crescentic glomerulonephritis than WT animals (50). Thus, they provided evidence that T_H_1 responses mediate severe crescentic injury and that T_H_1 and T_H_17 cells counter regulate each other during disease development in this model. In line with the *in vivo* observation, our group observed a skewing toward T_H_17 cells following *in vitro* stimulation of peripheral blood samples of AAV patients (51). Moreover, it has been shown that CD4^+^CD45RC^low^ cells (T cells with a memory phenotype) are a source of IL-17 in AAV patients (52). These observations were corroborated by Nogueira et al., demonstrating significant elevated levels of serum IL-17A and its associated upstream cytokine IL-23 in acute AAV patients (53). Additionally, auto-antigen-specific IL-17 producing cells were significantly elevated in patients during disease convalescence compared to healthy controls (53). Moreover, increased frequencies of circulating T_H_17 cells have been observed in various forms of vasculitis (GCA, EGPA, and Behçet disease) and correlated with disease activity (54–58).

A possible explanation for the involvement of T_H_17 cells in AAV lies within the major physiological role of T_H_17 cells. Physiologically, T_H_17 cells are important in the defense against fungi and bacterial infections [e.g., *Staphylococcus aureus (S.aureus)* infections] by activating neutrophils through the production of IL-17 and IL-17F. It has been shown that peptidoglycans and superantigens of *S. aureus* might have an immunomodulatory effect on DCs by imprinting of a strong T_H_17 polarization capacity (59). Furthermore, *S. aureus* α-toxin was shown to induce IL-17A secretion in CD4^+^ T cells (60). In addition, Zielinski et al. demonstrated that *S. aureus* specific T_H_17 cells produced IL-17 and surprisingly could produce IL-10 upon restimulation (61). Intriguingly, chronic nasal carriage of *S. aureus* has been found to be an important risk factor for disease relapse in AAV patients (62). This suggests that carriage of *S. aureus* may drive the T_H_17 responses in AAV.

The role of T_H_17 cells in atherosclerosis remains controversial. It has been demonstrated that T_H_17 cells and IL-17 accumulate in atherosclerotic lesion of both mice and humans, but both atherogenic as well as athero-protective effects of IL-17 have been reported (63–67). Studies in ApoE^−/−^ mice genetically deficient for IL-17 or treated with anti-IL-17A antibodies demonstrated that absence or depletion of IL-17 attenuated development of atherosclerosis (65, 68). Also, Ldlr^−/−^ mice transplanted with bone marrow from mice deficient in IL-17 receptor showed smaller atherosclerotic lesions (63). In patients, IL-17A expressing T cells were detected in atherosclerotic lesions and increased IL-17 expression in these lesions has been shown to be associated with increased inflammation and plaque vulnerability (67). In contrast to the pathogenic role of T_H_17 cells, Taleb et al. found a protective role for T_H_17 cells in atherosclerosis. Using Ldlr^−/−^ mice deficient for suppressor of cytokine signaling 3 (SOCS3), a suppressor of signaling from IL-17, showed less disease development (64). In the same study, administration of an anti-IL-17A antibody accelerated atherosclerosis, indicating a protective role for T_H_17 cells (64).

A possible explanation for these contradictory observations may be that IL-17 is not only produced by T cells. The presence of different IL-17 isoforms (IL-17A, -E, and -F) in human atherosclerotic plaques revealed that the IL-17 family cytokines were expressed by various cells of the immune system (e.g., neutrophils) depending on the stage of the atherosclerotic plaque (66). Furthermore, not only immune cell are targets of IL-17. It has been demonstrated that endothelial cells and smooth muscle cells are likely to be IL-17E-responsive, given the expression of IL-17 receptor components on these cells and transient activation of ERK1/2 upon stimulation with recombinant IL-17E (66). Thus, this indicates a complex contribution of IL-17 in atherogenesis depending on the isoform and phases of atherosclerosis.

Beside IL-17, T_H_17 cells can also produce IL-21, a cytokine that is produced primarily by T follicular helper (T_FH_) cells. IL-21 is required for B cell class switching, antibody production (69), and induces differentiation of B cells toward plasma cells by synergizing with B cell activating factor (BAFF) (70, 71). The role of IL-21 has been demonstrated in Behçet disease, a form of variable vessel vasculitis. Geri et al. demonstrated increased serum levels of IL-21 that correlated with the disease activity in patient with Behçet disease (58). In addition, they showed that IL-21 producing central memory CD4^+^ T cells positively correlated with T_H_17 responses and negatively correlated with FoxP3 T_REG_ cells. Conversely, blockade of IL-21 with IL-21R-Fc fusion protein resorted the balance between T_REG_ cells and T_H_17 cells by suppressing IL-17A production and increasing FoxP3 expression by CD4^+^ T cells (58). Interestingly, a significant increased population of IL-21 producing T_FH_ cells was observed in the circulation of AAV patients (72). In addition, IL-21 was shown to enhance the production of cytotoxic products such as granzyme B and perforin, by CD8^+^ T cells and natural killer (NK) cells (73). Based on the studies in various forms of vasculitis it is therefore conceivable that IL-21 together with IL-17 plays a critical role in the pathogenesis of AAV.

In atherosclerosis, no major studies have been conducted to date to investigate the role of T_FH_ cells, but IL-21 may be involved in tissue damage. There is evidence that IL-21 acts directly on gut epithelial cells to induce the production of macrophage inflammatory protein-3α (MIP-3α), a chemokine that attracts both T_H_1 and T_H_17 cells to inflamed tissues (74). Given that endothelial cells are known to produce MIP-3α, it is possible that IL-21 enhances the migration and accumulation of T_H_1 and T_H_17 cells into the vascular wall in both vasculitis and atherosclerosis resulting in inflammation.

### Regulatory T cells in vascular inflammation

The actions of T_H_ cells can be balanced by T_REG_ cells, a subpopulation that is characterized by their ability to suppress a variety of physiological and pathological immune responses and prevent autoimmunity (75). T_REG_ cells are characterized by their expression of forkhead/winged helix transcription factor (FoxP3) that is required for their development and function (76). Defects in T_REG_ function or reduced numbers of T_REG_ cells have been described in several autoimmune disorders and chronic inflammatory disorders associated with vascular inflammation (77). To date different research groups reported controversial results regarding the frequency of T_REG_ cells in AAV patients compared to healthy controls. However, a consistent finding has been that these studies all reported impaired functionality of circulating T_REG_ cells (78–80). It has been found that the suppressive function of T_REG_ cells was defective in GPA patients compared to healthy controls (78). However, the GPA patients showed a significant increase of memory FoxP3^+^CD25^high^ T_REG_ cells. Consistent with this finding, Klapa et al. demonstrated an increased number of FoxP3^+^ T cells as well as phenotypical and functional alteration of T_REG_ cells in GPA patients (79). They reported an increased number of interferon receptor I-positive T_REG_ cells in the peripheral blood of GPA patients. In addition, they showed that IFN-α exaggerates functional T_REG_ impairment *ex vivo* in response to the auto-antigen PR3 (79). Furthermore, Morgan et al. also reported altered T_REG_ function in GPA patients (80). They observed that T_REG_ cells from healthy controls and from ANCA-negative patients were able to suppress T cell proliferation to PR3, whereas T_REG_ cells from PR3-ANCA-positive patients failed to suppress this antigen specific response (80). Dysfunction of T_REG_ cells is thus believed to play a role in the development of GPA. In contrast, T_REG_ function in MPA patients was comparable to that in healthy controls although FoxP3 levels were diminished, suggesting that in MPA a numerical deficiency of T_REG_ cells exists (81). Additionally, Saito et al. demonstrated that the proportion of T_REG_ cells in the peripheral blood reflects the relapse or remission status of EGPA patients. They observed that FoxP3-expressing cells and IL-10 producing T_REG_ cells were detected in lower frequencies in patients with a relapse compared to patient in remission (82). However, the suppressive function of T_REG_ cells in EGPA patients still needs to be investigated. All together there are some inconsistent observations regarding the number and/or frequencies of T_REG_ cells in AAV patients. These differences might be due to variations in the methodology and gating strategies for the T_REG_ cells between the different studies. However, in all studies impaired functionality of the T_REG_ subset has been demonstrated indicating that T_REG_ cells from AAV patients are not able to suppress proliferation of other T_H_ cell subsets.

The terminally differentiated T_REG_ cells are not defined entirely by FoxP3 expression, and the FoxP3^+^ T cell population is heterogeneous, consisting of a committed T_REG_ lineage and an uncommitted subpopulation with developmental plasticity (83). It has been reported that human T_REG_ cells can convert into pro-inflammatory IL-17 producing T cells depending on a specific cytokine environment (81–83). Both T_H_ cell subsets (i.e. T_REG_ and T_H_17 cells) may develop from the same precursors under distinct cytokine conditions, and a subset of IL-17-producing CD4^+^FoxP3^+^ T_REG_ cells can be generated upon polarization by pro-inflammatory cytokines such as IL-6, which is crucial in orchestrating the balance of T_REG_ and T_H_17 cells (84–86). It has been shown that the combination of transforming growth factor-beta (TGF-β) and IL-6 treatment can synergistically promote FoxP3 degradation (87), and induce the transcription of ROR-γt, which in turn participates in the induction of IL-17 expression and mediates the skewing toward a T_H_17 cell phenotype.

Besides the local cytokine environment that orchestrates the balance of T_REG_ and T_H_17 cells, the functional stability of FoxP3 might influence the developmental pathway. Post-translational modifications can transiently alter the functionality of transcription factors, and there is evidence that FoxP3 can be regulated via acetylation. For example, hyperacetylation of FoxP3 increases the stability of FoxP3 and treatment with histone deacetylases inhibitors results in increased numbers and functional T_REG_ cells (88). Indeed, Koenen et al. demonstrated that histone deacetylases inhibitors suppresses the conversion from T_REG_ to T_H_17 cells (89). In addition, different isoforms of FoxP3 have been investigated in human T_REG_ that have been shown to affect T_REG_ function and lineage commitment. More specifically, the full length isoform FoxP3 interacts with ROR-γt and inhibits the expression of genes that define the T_H_17 lineage, whereas the isoform lacking exon 2, FoxP3Δ2 fails to inhibit ROR-γt. Upon stimulation in an inflammatory environment these non-functional T_REG_ convert into IL-17 producing effector T cells. Based on these findings, our previous described non-functional T_REG_ cells in AAV patients may lack their suppressive function due to the up-regulation of FoxP3Δ2 that fails to inhibit ROR-γt mediated IL-17 transcription. Indeed, Free et al., demonstrated that T_REG_ cells from patients with active AAV disproportionately used FoxP3Δ2, which might alter T_REG_ cell function (90).

In atherosclerosis, several studies have demonstrated a protective effect of T_REG_ cells. FoxP3^+^ T cells have been found in atherosclerotic plaques of humans, although in low numbers (91). In mice, the T_REG_ cytokine products IL-10 and TGF-β, have been demonstrated to induce potent anti-atherosclerotic activities. Genetic inactivation or blockade of IL-10 and TGF-β with neutralizing antibodies aggravated atherosclerosis in mice (92, 93). Depletion of T_REG_ cells directly addressed the protective role of these cells in atherosclerosis. Significant aggravation of atherosclerosis was observed in Ldlr^−/−^ mice with reduced T_REG_ cell numbers, achieved either by deletion of CD80/86 or CD28, inducible T cell co-stimulators, or upon treatment with CD25-depleting antibodies (94, 95).

Thus, the interplay and imbalances between different T_H_ cells are important in the pathogenesis of vascular inflammatory diseases (Table 1). An imbalance in T_H_1/T_H_2 toward the T_H_1 response promotes the development of vascular inflammation, whereas skewing toward prominent T_H_2 and T_REG_ responses is anti-inflammatory and results in a reduction of vascular inflammation. In AAV, T_H_17 cells are considered to be pathogenic but how T_H_17 cell affect inflammation in atherosclerosis still needs to be determined.

**Table 1:** **T cell subsets associated with vascular pathologies**.

T cell subset	Key characteristic	Finding in vascular pathology	Reference
T_H_1 cell	Production of IFNy	Skewing toward T_H_1 in localized GPA	(11, 28–30)
	Enhances cellular immune responses	Dominant T_H_1 cytokine prolife in human atherosclerotic plaques	(31)
T_H_2 cell	Production of IL-4	Skewing toward T_H_2 in active generalized GPA, and in EGPA	(28, 29)
	Promote humoral immune response		
T_H_17 cell	Production of IL-17	Skewing toward T_H_17 in GPA during quiescent disease, and in EGPA and Behçet disease during active disease	(51, 57, 58)
	Defense against fungi and bacterial infections		
	Mediates pathogenic responses in autoimmune diseases	Increased frequencies of T_H_17 cells in GCA	(54–56)
		Contradictory observations of T_H_17 cell function in atherosclerosis, IL-17A expressing T cells are present in human atherosclerotic lesions and associated with increased inflammation and plaque vulnerability. However, mouse models reveal a protective effect of T_H_17 cells	(63–67)
T_FH_ cell	Production of IL-21	Increased T_FH_ population in GPA	(72)
	IL-21 required for B cell class switching	T_FH_ cytokine IL-21 correlates with disease activity in Behçet disease	(58)
T_REG_ cell	Production of IL-10	Contradictory observations regarding the frequency of T_REG_ cells in GPA patients, however all studies report an impaired function of T_REG_ cells in GPA	(78–80, 90)
	Regulation of other T cell subsets		
	Maintain peripheral tolerance to self antigens		
		Numerical defect of T_REG_ cells in MPA	(81)
		T_REG_ cells reflect relapse and remission status in EGPA	(82)
		Reduced frequencies of T_REG_ cells in GCA	(55, 56)
		Low number of T_REG_ cells in human atherosclerotic plaques, mouse models reveal a protective effect of T_REG_ cells	(91–93)

*GPA, granulomatosis with polyangiitis; EGPA, eosinophilic granulomatosis with polyangiitis; GCA, giant cell arteritis; MPA, microscopic polyangiitis*.

### Involvement of CD4^+^ T effector memory cells

As mentioned above, several observations support the involvement of CD4^+^ T_H_ cells in the pathogenesis of vascular inflammatory diseases like AAV and atherosclerosis. In line with these observations, an expanded population of CD4^+^ T cells lacking the co-stimulatory molecule CD28 was observed in peripheral blood and in inflammatory lesions of AAV patients (10, 96). Furthermore, these CD4^+^CD28^−^ T cells are a major source of IFN-γ and TNF-α, display up-regulation of the T cell differentiation marker CD57 and show cytoplasmic perforin expression, indicating the cytotoxic potential of these cells (10).

Consistent with these findings, our group observed a significant increase in the frequency of circulating CD4^+^ T_EM_ cells in the peripheral blood of AAV patients in remission (97). Subsequently, it was found that the number of these circulating CD4^+^ T_EM_ cells decrease during active disease when compared with the number during complete remission (97). We proposed that these CD4^+^ T_EM_ cells migrate toward inflamed tissues (97). In accordance, infiltrating T cells found in granulomas within lung and/or kidney tissues resemble mainly the CD4^+^ T cell memory phenotype. A remarkable increase in CD4^+^ T_EM_ cells in the urinary sediment with a concomitant decrease of circulating CD4^+^ T_EM_ cells in patients with active renal involvement strongly suggests migration of CD4^+^ T_EM_ cells during active renal disease into the affected organs (98). This finding might reflect the role of CD4^+^ T_EM_ cells in renal injury during active disease. In line with these findings, is the observation that CD4^+^ T_EM_ cells expressing CD134 are expanded in peripheral blood of AAV patients (99). It has been reported that the ligand for CD134 (CD134L) is expressed on endothelial cells (100) and ligation of CD134 contributes to T cell migration and tissue infiltration through its interaction with CD134L on vascular endothelial cells (101). Furthermore, CD134 expressing T cells have been detected in the inflammatory lesions of AAV patients (99), supporting the hypothesis that CD4^+^ T_EM_ migrate to inflamed areas. The fact that CD4^+^ T_EM_ cells migrate to the kidney during active disease suggests that a specific stimulus is being expressed by the (micro)vascular bed of the kidney, which attracts these cells.

In atherosclerotic disease, analysis of CD4^+^ T cell subsets (i.e., naïve T cells, T_CM_, and T_EM_ cells) revealed that CD4^+^ T_EM_ cells are key players in disease pathogenesis (102). It has been shown that the frequency of circulating T_EM_ cells was significantly increased in Ldlr^−/−^ and ApoE^−/−^ mice compared to control C57BL/6 mice and also correlated with the extent of atherosclerotic lesions (102). In line with these observations, Almanzar et al. demonstrated that T cells isolated from early atherosclerotic lesions are mostly CD4^+^ T_EM_ cells (24). Subsequently, the intralesional atherosclerotic CD4^+^ T cells produce high amounts of the pro-inflammatory cytokines IFN-γ and IL-17 (24), which suggest that memory T cells in atherosclerotic plaques are in a state of activation reflecting the pro-inflammatory cytokine production.

At the functional level, CD4^+^ T_EM_ cells have been shown to mimic features of NK cell including surface expression of the NK group 2 member D (NKG2D) and cytotoxic potential (103, 104). NKG2D is an activating C-type lectin-like receptor, which differs from other NKG2 family members as it apparently lacks an antagonist and substitutes for CD28-mediated co-stimulatory signaling in CD28^−^ T_EM_ cells (104). One of the NKG2D ligands in human is the major histocompatibility complex class-I chain-related molecule A (MICA). MICA is usually absent on normal cells, but expressed upon cellular stress on target cells such as fibroblasts, epithelial cells, and endothelial cells (104). The expression of MICA on the surface of the endothelium makes this polymorphic molecule a potential target in vasculitis. In rheumatoid arthritis (RA), an other chronic and systemic autoimmune disorder, an unusual CD4^+^CD28^−^NKG2D^+^ population was detected in peripheral blood and synovial tissue (105). Furthermore, the NKG2D ligand MICA is dramatically upregulated in RA synoviocytes and is capable of activating autoreactive T cells in an NKG2D-dependent manner (105). In addition, it has been shown in patients with Crohn’s disease, a chronic inflammatory disorder, that CD4^+^NKG2D^+^ T_EM_ cells can kill target cells that express MICA via NKG2D–MICA interaction (106). These findings in RA and Crohn’s disease translate very well to AAV. In AAV, it has been found that NKG2D was anomalously expressed on circulating CD4^+^CD28^−^ T_EM_ cells (107). Furthermore, it has been demonstrated that NKG2D, MICA, and IL-15 are simultaneously expressed in granulomatous lesions in AAV patients. Importantly, it was reported that survival, expansion, and cytotoxic properties of CD4^+^NKG2D^+^ T cells were dependent on IL-15 signaling in AAV (108). Therefore, it is tempting to speculate that combined IL-15 and MICA expression contributes to the killing mechanisms of CD4^+^NKG2D^+^ T cells in vessel inflammation and disease progression in AAV.

In atherosclerosis, Xia et al. reported that immune activation resulting from NKG2D–ligand interaction promotes atherosclerosis (109). They observed soluble MICA in sera and upregulated MICA expression in atherosclerotic plaques of patients with type 2 diabetes mellitus. Moreover, they investigated the role of NKG2D in atherosclerosis using ApoE^−/−^ mice genetically deficient for NKG2D or treated with anti-NKG2D antibodies. Preventing NKG2D–ligand interaction resulted in a dramatic reduction in plaque formation and suppressed systemic and local inflammation mediated by multiple immune cell types. Since this is the only study reported on NKG2D in relation with atherosclerosis development, further studies are needed to fully elucidate the role of NKG2D in the pathogenesis of atherosclerosis.

## T Cell Directed Therapeutic Interventions

As described in this review, T cells and T cell migration are pivotal in vascular inflammatory diseases such as vasculitides and atherosclerosis. Therefore interfering with T cell activation, proliferation, and migration might be a beneficial approach to dampen the inflammatory response and cell-based therapy to modulate the T cell compartment may be a therapeutic option.

Regulatory T expansion may be of benefit to counterbalance persistent T cell activation in AAV. In this respect, treatment with low-dose IL-2 has been found to promote T_REG_ recovery and clinical improvement in patients with autoimmune vasculitis (110). Interestingly, tocilizumab, a humanized anti-IL-6 receptor antibody used in the treatment of RA demonstrated that blocking IL-6 function affects the balance between T_H_17 and T_REG_ cells favoring a more anti-inflammatory response (111). In addition, control of T cell activation might be an attractive therapeutic possibility. In this regard, blockage of the co-stimulatory pathway CD28/CD80 using CTLA-4 fusion proteins has successfully been used in RA (112) and shown to be well tolerated in a small open-label trial in GPA patients (113), which suggests this treatment as a possible therapeutic opportunity in AAV.

Modulation of other T cell subsets is also considered as a means for future therapies. For example, since T_H_17 cells contribute to inflammation and granuloma formation, this T_H_ cell subset could be a novel therapeutic target for AAV. Depletion of T_H_17 cells by targeting specific surface proteins may be difficult as T_H_17 cells share many surface markers with other T cell subsets. Another therapeutic approach could be to specifically target its signature cytokine, IL-17, which would probably be more feasible. Indeed, neutralizing IL-17 by IL-17A specific antibodies or administration of soluble IL-17 receptors reduces inflammation in animal models of atherosclerosis (49, 65, 68, 114). Several IL-17A blockers, including the anti-IL-17A monoclonal antibodies secukinumab and ixekizumab, and the anti-IL-17 receptor subunit A monoclonal antibody brodalumab have been evaluated in clinical trials (115–117), and shown to induce clinically relevant responses in patients. Besides IL-17, IL-21 also seems an interesting target in the treatment of autoimmune mediated vascular inflammation. Manipulation of IL-21 levels may have desirable therapeutic consequences as it might reduce the recruitment of inflammatory T_H_1 and T_H_17 cells to inflammatory lesions preventing tissue damage and inhibit expansion of autoreactive B cells. Recently, phase I clinical trials using an IL-21-specific monoclonal antibody have been completed for RA (NCT01208506 and EudraCT-2011-005376-42, www.clinicaltrial.gov) but terminated for SLE (NCT01689025, www.clinicaltrial.gov). Neutralization of IL-17 or IL-21 could therefore represent also novel therapeutic approaches for patients with AAV. However, experiments using animal models of AAV and clinical trials need to elucidate the therapeutic potential of neutralizing IL-17 and IL-21 in AAV. It is important to note that interfering with the cytokine environment might also cause disturbances in the developmental pathways of the different T cell lineages. Since there is a tight interplay between different T cell lineages such as the T_REG_ and T_H_17 cells, one should be cautious in modulating the cytokine environments. Besides targeting pro-inflammatory cytokines like IL-17 or IL-21, one can also consider to target effector T cells, which are predominantly responsible for the production of these cytokines.

### Effector memory CD4^+^ T cells as therapeutic targets

According to aforementioned evidence in AAV and atherosclerosis, CD4^+^ T_EM_ cells are considered to play a pivotal role in the pathogenesis of vascular inflammation and therefore, may serve as a potential therapeutic target. Selective targeting of CD4^+^ T_EM_ cells without impairing other parts of the humoral or cellular immune system could be a major step forward in the treatment of chronic and/or autoimmune mediated vascular inflammation disorders.

The capacity of CD4^+^ T_EM_ cells to interact with target cells via NKG2D–MICA interaction and attack them by releasing cytolytic enzymes has been demonstrated (104, 106). Therefore, NKG2D expressed on pathogenic T_EM_ cells could be an interesting target to inhibit the pathogenic effects of T_EM_ cells. Interestingly, interference with NKG2D signaling using anti-NKG2D antibodies has shown beneficial effects when administered early in two different mouse models. Blockade of NKG2D prevented autoimmune diabetes in non-obese diabetic mice (118) and attenuated transfer-induced colitis in SCID mice (119). However, no clinical trials on the blockade of NKG2D or its ligands in autoimmune diseases have been conducted.

Besides interfering with the NKG2D–MICA interaction, biophysical analyses revealed that ion channels expressed by immune cells perform functions vital for cellular homeostasis and T cell activation [reviewed by Ref. (120)]. In particular, human T cells express two types of potassium channels (voltage-gate potassium Kv1.3 channel; Kv1.3 and Ca^2+^-activated potassium KCa3.1 channel; KCa3.1) that play a major role in their activation. Interestingly, Kv1.3 channels and the KCa3.1 channels are expressed on T cells in a distinct pattern that depends on the state of activation as well as on the state of differentiation of the given T lymphocyte subset (121). It has been shown that Kv1.3 channels are highly expressed in CD4^+^ T_EM_ cells (~1500 channels per cell), whereas naïve and T_CM_ cells express lower levels of Kv1.3 channels (~250 channels per cell) (122). Therefore, Kv1.3 channels may serve as an attractive target for specific immunomodulation in T_EM_ cell mediated chronic or autoimmune diseases. Hu et al. have demonstrated that genetic silencing of Kv1.3 in human CD4^+^ T cells results in selective expansion of T_CM_ cells and the disappearance of T_EM_ cells after multiple rounds of stimulation with anti-CD3/CD28 *in vitro*, suggesting that Kv1.3 is essential for maintaining the T_EM_ pool (123). Indeed, selective blocking of Kv1.3 channels inhibits Ca^2+^ signaling, pro-inflammatory cytokine production, and proliferation of CD4^+^ T_EM_ cells *in vitro*, with little or no effects on CD4^+^ naïve and T_CM_ cells (124). Furthermore, it has been shown that specific Kv1.3 blockade suppressed T_EM_ cell motility in inflamed tissues, but had no effect on homing to or motility in lymph nodes of naïve and T_CM_ *in vivo* (125). In addition, Kv1.3 blockers ameliorate disease development in animal models of multiple sclerosis, RA, T1DM, and contact dermatitis without compromising the protective immune responses to acute infections (124, 126, 127). Noteworthy, Gocke et al. demonstrated that genetic deletion of Kv1.3 biases T cells toward an immunoregulatory phenotype and renders mice resistant to experimental autoimmune encephalomyelitis (128). They showed that Kv1.3 is required for expression of pro-inflammatory cytokines IFN-γ and IL-17, whereas its absence led to increased IL-10 production (128). Thus, it is tempting to speculate that pharmacological blockade or genetic suppression of Kv1.3 channels can be employed as a means to skew CD4^+^ T cell differentiation toward a regulatory phenotype, which might be beneficial for autoimmune mediated vascular diseases in general. Importantly, Kv1.3 blockers have a good safety prolife in rodents and primates and do not compromise the protective immune response to acute viral (Influenza) or bacterial (*Chlamydia*) infections (124, 125, 127).

Taken together, these studies demonstrated that specific blockade of Kv1.3 channels on T_EM_ cells suppresses the pathogenicity of T_EM_ cells by inhibition of their activation, proliferation and migration. Recently, it has been shown that expression of T cell Kv1.3 channels correlated with disease activity in ulcerative colitis (129). Therefore, Kv1.3 channels on CD4^+^ T_EM_ cells in chronic or autoimmune inflammatory diseases could constitute a novel pharmacological target in immunomodulation therapies and at the same time may serve as a marker for disease activity.

## Conclusion

Vascular inflammation can be driven by chronic inflammatory disorders or by autoimmune mediated diseases. In this inflammation process, there is a tight interplay between the innate and adaptive immune system. However, as described in this review, substantial evidence point to an important role for cell mediated adaptive immune responses in the pathogenesis of vascular inflammation. In particular, T cells in chronic or autoimmune mediated vascular inflammation show several functional abnormalities. The T cell compartment shows dysregulation in T_H_ subsets, including an imbalance between T_H_1 and T_H_2 cells, skewing toward T_H_17 response and defective functions of T_REG_ cells. However, the importance of T_H_2 and T_H_17 cells in atherogenesis is controversial. The underlying mechanisms responsible for orchestrating the aberrations within the T cell compartment are not completely understood. However, multiple studies indicate an interplay between T_REG_ cells and T_H_17 cells. It has been suggested that in the context of an inflammatory environment T_REG_ cells convert into IL-17 producing cells. Furthermore, T_REG_ cells clearly have a protective effect in experimental models of atherosclerosis. In AAV, T_REG_ cells are often quantitatively or functional defective. It has been suggested that a defect in T_REG_ cell function, may also contribute to the expansion of CD4^+^ T_EM_ cell population and migration of these cells to inflamed sites. Indeed, observations in AAV and atherosclerosis support T_EM_ cell involvement in vascular inflammation that in part contributes to tissue damage. The persistent activation of T_EM_ cells results in a selective up-regulation of Kv1.3 channels. These potassium channels play a key role in T_EM_ cell homeostasis, proliferation, and activation. Therefore, they seem to be a highly interesting target for immunomodulation of T_EM_ cells without compromising other subsets of the T cell compartment.

Currently efforts are being made to develop biological agents that can modulate the different T cell compartments specifically. In the case of autoimmune mediated vascular diseases such strategies could first be used for the prevention of disease flares. Considering that expansion of T_REG_ cells might be inadequate to control inflammatory responses, regulating T_EM_ cells would probably be the most effective approach. This approach may diminish effector responses and convert these into a more disease regulating response. To develop such therapeutic strategies further studies on the basic immunological properties of T_EM_ and T_REG_ cells in especially humans are needed. Investigation of the functional characteristics of T_EM_ cells in the pathogenesis of vascular inflammatory diseases and selective targeting of these cells will enable their application for the treatment of T_EM_ cell mediated vascular diseases.

## Conflict of Interest Statement

The authors declare that the research was conducted in the absence of any commercial or financial relationships that could be construed as a potential conflict of interest.
